# Bibliometric analysis of human microbiota-associated animal model (2005–2025)

**DOI:** 10.3389/fmicb.2026.1777297

**Published:** 2026-04-30

**Authors:** Xiangning Huang, Xinyu Yang, Yunfeng Yu, Jiawang Huang, Wang Tao, Rong Yu

**Affiliations:** 1 School of Traditional Chinese Medicine, Hunan University of Chinese Medicine, Changsha, China; 2 Department of Endocrine, The First Hospital of Hunan University of Chinese Medicine, Changsha, China

**Keywords:** animal model, bibliometric analysis, fecal microbiota transplantation, human microbiota-associated animal models, microbiota

## Abstract

**Background:**

The research on human microbiota-associated (HMA) animal models is an important tool for studying the human microbiome and holds great potential for elucidating disease mechanisms and microbe-based therapeutic interactions. However, a systematic bibliometric assessment of this field has been limited.

**Methods:**

This study employed bibliometric methods, retrieving relevant publications published between 2005 and 2025 from the Web of Science Core Collection, Scopus, and PubMed, and visualizing the data with VOSviewer and CiteSpace.

**Results:**

The analysis revealed a continuous upward trend in the number of publications on this topic. The United States and its research institutions contributed the most and maintained close collaborations with multiple countries. The majority of the articles appeared in journals such as *Gut Microbes, Microbiome*, and *Proceedings of the National Academy of Sciences of the United States of America (PNAS)*. Keyword and highly-cited reference analyses focused on the application of these models in investigating disease mechanisms and therapeutic exploration, particularly for metabolic, gastrointestinal, oncological, and neurodegenerative diseases. In addition, the impact of modeling factors such as diet and host genetics on the models has also attracted attention.

**Conclusion:**

HMA animal models have become a core platform linking clinical and basic microbiology research, demonstrating unique advantages in recapitulating disease-associated microbial features and phenotypes. Nevertheless, because these models are essential for testing causal links between microbiota and disease, methodological standardization and procedural refinement are needed to enhance reproducibility and clinical applicability.

## 1 Introduction

Growing interest has been directed toward the correlation between the human microbiota and overall health. Moreover, prior research has demonstrated links between the intestinal microbiome and the development of numerous conditions such as obesity, type 2 diabetes, non-alcoholic fatty liver disease, malnutrition, and cancer (Fan and Pedersen, 2021; Wong and Yu, 2023). However, the majority of investigations only examine compositional differences in microbiota between patients and healthy controls, making it difficult to determine whether dysbiosis is a cause, a consequence, or merely concurrent with disease (Brüssow, 2020; Lv et al., 2021). In order to more convincingly clarify the causality between microbiota and disease, researchers have continually experimented with and designed novel animal models (Fan and Pedersen, 2021). Among these, fecal microbiota transplantation (FMT) derived “human microbiota-associated (HMA) animal models” are considered the most persuasive tools (Aguanno et al., 2022). Introducing human microbiota directly into germ-free (GF) or antibiotic-treated hosts enables HMA animal models to reproduce the donor’s micro-ecological features within the animal (Kostic et al., 2013). Such models allow researchers to examine the impact of particular microbial taxa on host metabolic, immune, and neurological phenotypes, thereby providing experimental validation of the causal relationship between microbes and disease (Marcobal et al., 2013; Ridaura et al., 2013; Planer et al., 2016; Sharon et al., 2019).

Despite the considerable promise of HMA animal models for establishing causality, there remains an absence of comprehensive building protocols and bibliometric evaluations. Existing *Guidelines for reporting animal fecal transplantation studies* (Secombe et al., 2021) and *Clinical Practice Guideline on Fecal Microbiota-Based Therapies* (Cammarota et al., 2017; Peery et al., 2024) are primarily directed at intra-species applications and lack a standardized framework that addresses the methodological nuances of HMA animal models. Bibliometric investigations have largely focused on disease-specific microbial correlations or the general clinical use of FMT, and systematic examinations of the scholarly output, collaborative networks, and research trends of HMA animal models remain scarce. Consequently, the current state of research and emerging hotspots concerning HMA animal models remain ambiguous, creating a notable void in the discipline. Accordingly, we conducted a systematic bibliometric review of publications centered on HMA animal models to elucidate research trends, key institutions, thematic hotspots, and collaborative structures. The aim of this work is to propel the development of human microbiota research in disease mechanisms and therapeutic applications.

## 2 Materials and methods

### 2.1 Data sources and search strategy

To maximize both coverage and quality of the dataset, we employed several scholarly databases as our sources. In this study, the data were drawn from the Web of Science Core Collection (WoSCC), Scopus, and PubMed. WoSCC is notable for its broad disciplinary coverage, spanning the natural sciences, social sciences, and humanities, making it an excellent database choice for interdisciplinary research. Scopus, a globally recognized abstract-citation database, indexes a vast amount of scholarly resources, including numerous journals and conference proceedings. PubMed supplies an extensive corpus of health and medical-oriented publications, serving as a pivotal resource for biomedical research.

We applied a Boolean retrieval strategy across the aforementioned databases with the query: TS = (human* (microbiota OR microbiome OR flora OR gut microbiota OR gut flora) AND (animal* OR mouse* OR rat* OR rodent* OR pig* OR host OR organism) AND (gnotobiotic OR germ-free OR axenic OR antibiotic-Induced) AND (associat* OR transplant* OR coloniz* OR humaniz*)). The search period was limited from 1 January 2005 to 1 October 2025, and only English-language records were included.

### 2.2 Inclusion and exclusion criteria

To guarantee both the quality and validity of the data sources, this study applied the following inclusion and exclusion criteria to the selected literature. Inclusion criteria: (1) relevance to the research topic; (2) the literature types only include research papers and review papers. Exclusion criteria: (1) retracted publications; (2) duplicate records; (3) conference papers, book chapters, and similar items.

### 2.3 Literature screening

Based on the aforementioned query, the search results were saved as BibTeX, CSV, and plain-text files to facilitate later selection and analysis. The literature was independently screened by two investigators (XH and XY) according to the predefined inclusion and exclusion criteria. Initially, duplicates were eliminated through verification of DOIs and article titles. Subsequently, articles unrelated to the study theme were discarded after reviewing titles and abstracts. For publications lacking an abstract, the full text was downloaded and examined for eligibility. The investigators then cross-validated their screening results, and disagreements were resolved by mutual agreement. Finally, the confirmed articles were compiled into CSV and plain-text files for subsequent analytical work.

### 2.4 Data collection and analysis

The data described above were loaded into Microsoft Excel 2021 to organize the information, construct a bibliometric database for the HMA animal models study, and generate publication-trend visualizations. The subsequent data analysis and visualization employed the following applications: (1) VOSviewer (v1.6.20) (Van Eck and Waltman, 2010), which generates bibliometric maps from the co-occurrence matrices. (2) Scimago Graphica (v1.0.49) (Hassan-Montero et al., 2022), a code-free visualization platform using JSON, capable of swiftly creating interactive figures and exporting them in multiple file types. (3) CiteSpace (v6.4.R1), a scientific knowledge-graph application created by Drexel University, and the most popular software for bibliometric analysis and visualization. (4) Bibliometrix (R package, version compatible with R 4.5.1) (Aria and Cuccurullo, 2017), an open-source bibliometric toolkit providing an end-to-end pipeline covering literature import, co-citation networks, keyword co-occurrence, and scientific mapping, frequently used together with Biblioshiny for interactive exploration. VOSviewer and Scimago Graphica were used to conduct co-citation analysis and to visualize collaborative networks of countries/regions, institutions, and authors. CiteSpace was used to perform keyword-cluster analysis and burst detection, facilitating the identification of research frontiers and trends. Moreover, we integrated Bibliometrix to carry out author citation-weight index calculations, journal and high-citation article analyses, and to depict country/region collaboration networks.

## 3 Results

### 3.1 Literature screening process

**FIGURE 1:**
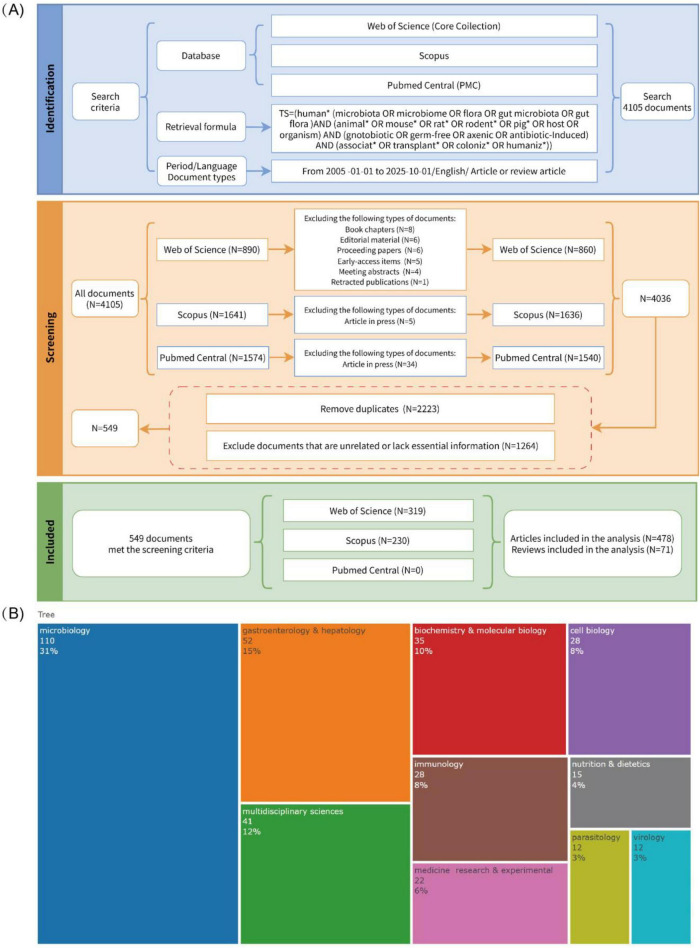
Flow diagram of the literature screening and research field overview. **(A)** Flow diagram of the literature screening. **(B)** Research field overview.

As shown in Figure 1A, we retrieved 890, 1,641, and 1,574 records from WoSCC, Scopus, and PubMed, respectively, totaling 4,105 publications. After excluding document types such as book chapters, editorial material, proceeding papers, early-access items, meeting abstracts, and retracted publications, we merged the three databases and retained 4,036 records suitable for further analysis. Applying our predefined inclusion and exclusion criteria, we meticulously screened the records for relevance and completeness. This rigorous vetting process led to the removal of 2,223 duplicate entries and 1,264 publications that were either thematically irrelevant or lacked essential information. Consequently, our final dataset comprised 549 publications, including 478 articles and 71 reviews. These works involve 3,775 authors from 808 institutions across 50 countries and regions, were published in 231 journals, and cite a total of 17,205 references. Figure 1B shows that studies on the HMA animal models are concentrated in the fields of microbiology (110 articles, 31%), gastroenterology and liver disease (52 articles, 15%), and interdisciplinary research (41 articles, 12%).

### 3.2 Trend of publication output

**FIGURE 2:**
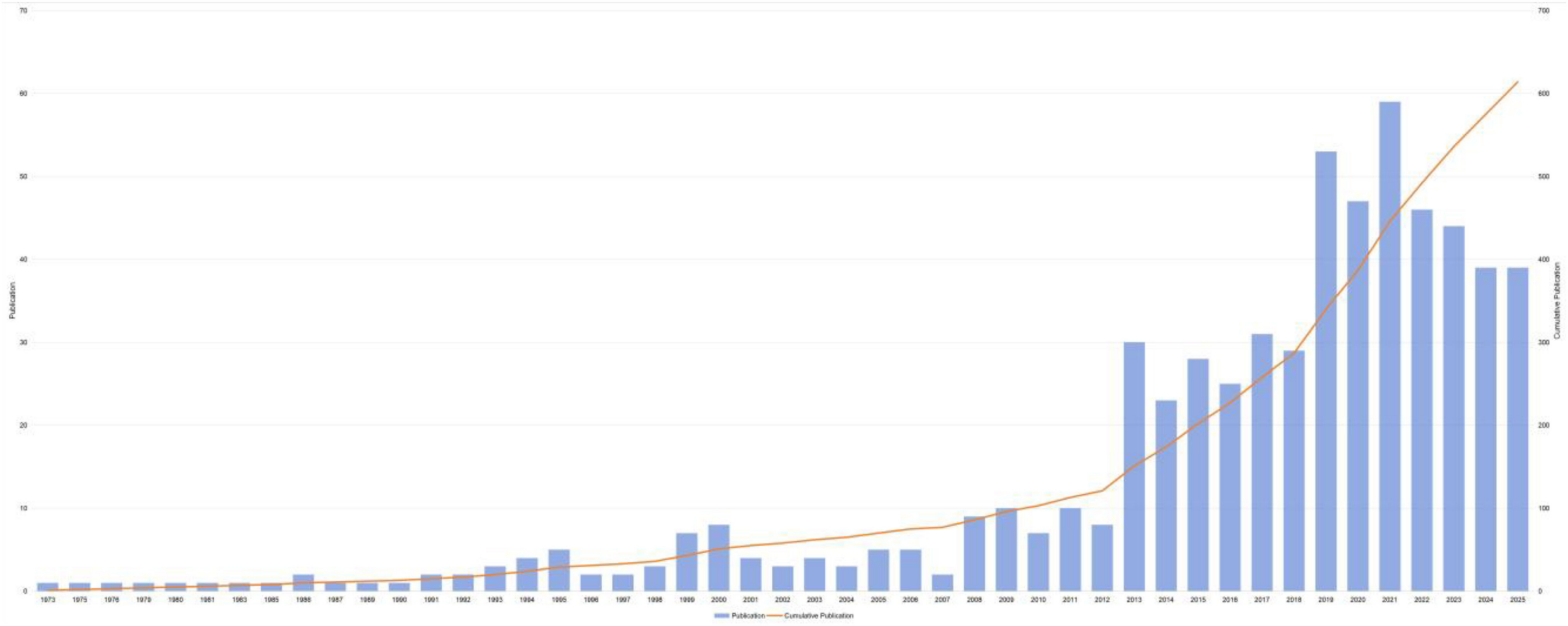
Trends of the annual publication output. Temporal trend of annual publication output and cumulative publications from 2005 to 2025.

As shown in Figure 2, the publication output of this field remained stable from 2005 to 2012. After 2012, a pronounced surge was observed: only eight papers were published in 2012, compared with 30 in 2013. In 2021, the output peaked at 59 papers and has been maintained ever since. The interval from 2019 to 2025 represents a high-productivity phase, comprising 59.56% of all publications. Meanwhile, the cumulative publication count exhibits exponential growth, indicating expanding research interest and scholarly output in the field. Overall, the trend suggests that the field is undergoing rapid development and is likely to continue to exhibit strong growth potential in the coming years. This trend also signals sustained, increasing attention and investment from the scholarly community, furnishing a solid foundation for future related studies.

### 3.3 Countries/regions distribution and collaboration

**FIGURE 3:**
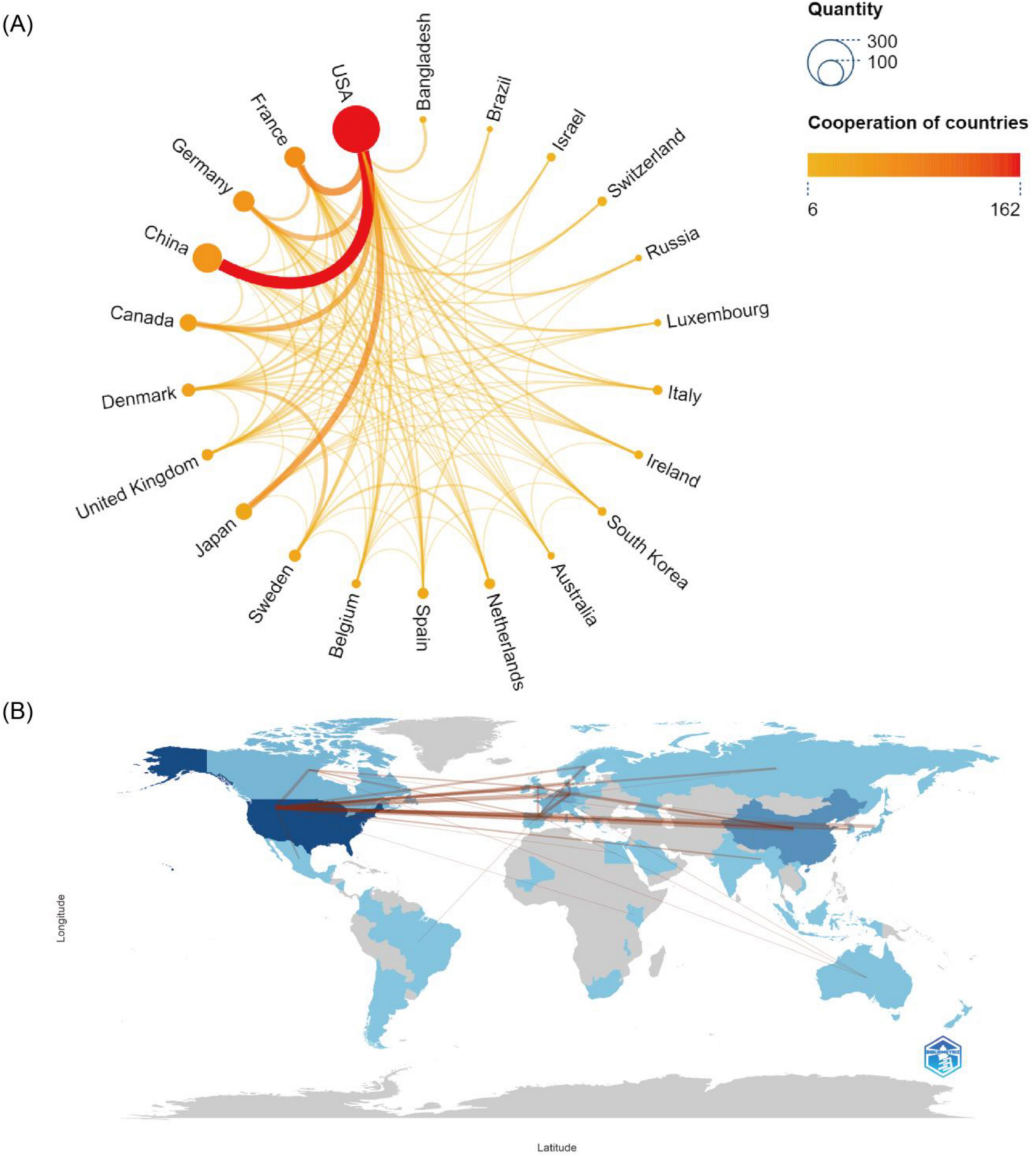
Visualization of country/region distribution and collaboration. **(A)** Circle diagram of countries/regions distribution and collaboration. The size of the circle nodes is proportional to the number of publications from each countries/regions, while the thickness and color of the connecting lines represent the strength of collaboration. **(B)** World map of countries/regions distribution and collaboration. The thickness of the connecting lines indicates the degree of collaboration between countries/regions, and the depth of color represents the publication volume.

Table 1 shows that the United States (USA) (288 papers, 37.21 %) leads the world in publications on this topic, followed by China (112 papers, 14.47 %), Germany (58 papers, 7.49 %) and France (56 papers, 7.24 %). All other countries each contribute < 5% of the total output. The average citations per item (ACI) is defined as the mean number of citations per paper, calculated by dividing the total citation count by the number of documents, and serves as an effective indicator of citation impact. France exhibits the highest ACI (407.77), followed by Sweden (384.05), Denmark (276.61) and the USA (232.70). Although China ranks second in publication count, its ACI is only 96.71, markedly lower than the aforementioned nations. Centrality evaluates the topological significance of network nodes, and in CiteSpace, nodes with betweenness centrality values of at least 0.1 are classified as key nodes. Only the USA (1.06) and Germany (0.26) surpass the 0.1 threshold, underscoring their pivotal roles. Country/region collaboration chord diagrams and network maps were generated using Scimago Graphica and Bibliometrix, as illustrated in Figure 3. The USA, which has the highest publication output, maintains strong collaborative ties with China, France, Germany, Canada and Japan, with China being its closest partner.

**TABLE 1:** Top 10 productive countries/regions.

Rank	Country	Quantity	Proportion/%	Citations	ACI	Centrality
1	USA	288	37.21%	67,019	232.70	1.06
2	China	112	14.47%	10,832	96.71	0.04
3	Germany	58	7.49%	4,452	76.76	0.26
4	France	56	7.24%	22,835	407.77	0.04
5	Canada	38	4.91%	5,496	144.63	0.07
6	Japan	36	4.65%	6,071	168.64	0.09
7	Denmark	23	2.97%	6,362	276.61	0.01
8	Sweden	19	2.45%	7,297	384.05	0.01
9	United Kingdom	17	2.20%	3,449	202.88	0.04
10	Spain	16	2.07%	2,272	142.00	0.1

ACI, average citations per item.

### 3.4 Institutions distribution and collaboration

Table 2 lists the 10 most productive institutions out of a pool of 808 worldwide entities conducting HMA animal models research. Six are based in the USA, two in China, and the other two in Denmark and Canada. Washington University School ranks first in publication quantity, citations, and ACI; its 38 papers have garnered 25,808 citations (ACI = 679.16), an outstanding result.

**TABLE 2:** Top 10 productive institutions.

Rank	Institution	Country	Quantity	Citations	ACI
1	Washington University School	USA	38	25,808	679.16
2	The Ohio State University	USA	12	240	20.00
3	University of Copenhagen	Denmark	12	2,439	203.25
4	Shanghai Jiao Tong University	China	11	1,274	115.82
5	Stanford University	USA	11	3,747	340.64
6	The University of Chicago	USA	11	2,951	268.27
7	Mayo Clinic	USA	10	2,185	218.50
8	McMaster University	Canada	10	2,721	272.10
9	The University of North Carolina System	USA	10	480	48.00
10	Chongqing Medical University	China	9	2,032	225.78

ACI, average citations per item.

**FIGURE 4:**
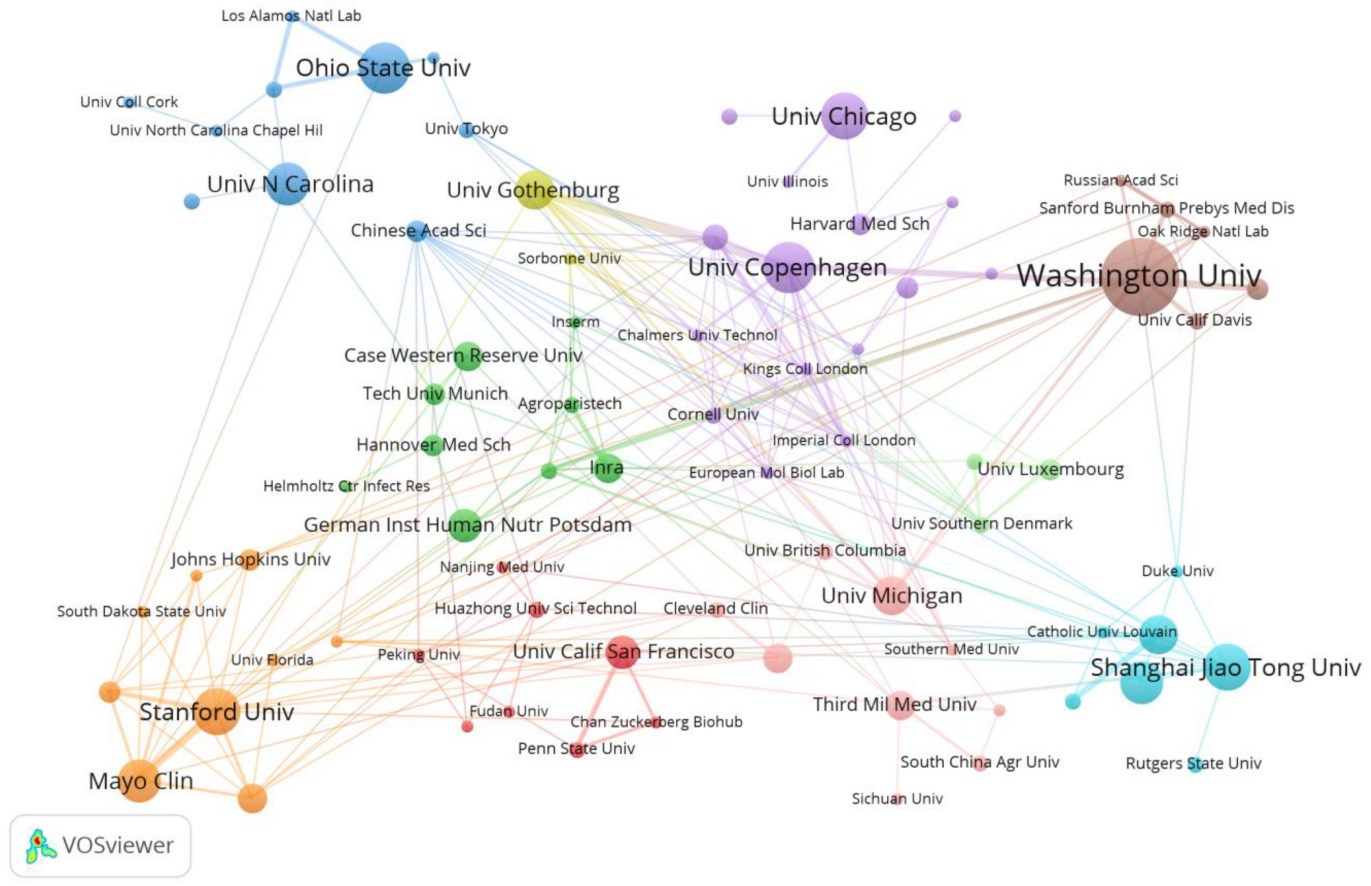
Visualization of institutions’ distribution and collaboration. Nodes represent research institutions, and their size reflects the number of articles published. Node colors correspond to different research regions or countries. Color gradients and the thickness of connecting lines indicate the research density and strength of connections within each region.

Figure 4 shows the VOSviewer generated institutional co-occurrence map, illustrating inter-institutional collaborations and research activity levels. Among the top—10 institutions, the collaboration between Washington University School and the University of Copenhagen is the most intensive, with the next-strongest link observed between Mayo Clinic and Stanford University.

### 3.5 Authors distribution and collaboration

A total of 3,775 authors worldwide have published literature related to this topic. Table 3 presents the publication volume, H-index, and ACI for the 10 most prolific authors. Among them, six authors are from the USA, two from China, and two from Germany. The three leading authors in terms of both publication output and H-index are Jeffrey I. Gordon (USA; 26 papers, H-index = 22), Wei Hong (China; 19 papers, H-index = 13), and Blaut Michael (Germany; 15 papers, H-index = 13). The highest ACI values belong to Jeffrey I. Gordon (USA; ACI = 676.42), Rey, Federico E (USA; ACI = 597.46), and Zeng Benhua (China; ACI = 226.89).

**TABLE 3:** Top 10 authors.

Rank	Author	Country	Institution	Quantity	Citations	ACI	H-index
1	Jeffrey I. Gordon	USA	Washington University	26	17,587	676.42	22
2	Wei, Hong	China	Sun Yat-sen University	19	794	41.79	13
3	Blaut, Michael	Germany	German Institute of Human Nutrition Potsdam-Rehbrucke	15	1,112	74.13	13
4	Rey, Federico E	USA	University of Wisconsin	13	7,767	597.46	13
5	Sartor, R. Balfour	USA	University of North Carolina	11	636	57.82	10
6	Cheng, Jiye	USA	Washington University	10	1,843	184.30	9
7	Rajashekara, Gireesh	USA	Ohio State University	10	226	22.60	9
8	Zeng, Benhua	China	Third Military Medical University	9	2,042	226.89	9
9	Hibberd, Matthew C	USA	Washington University	9	994	110.44	8
10	Loh, Gunnar	Germany	German Institute of Human Nutrition Potsdam-Rehbrucke	9	826	91.78	9

ACI, average citations per item.

**FIGURE 5:**
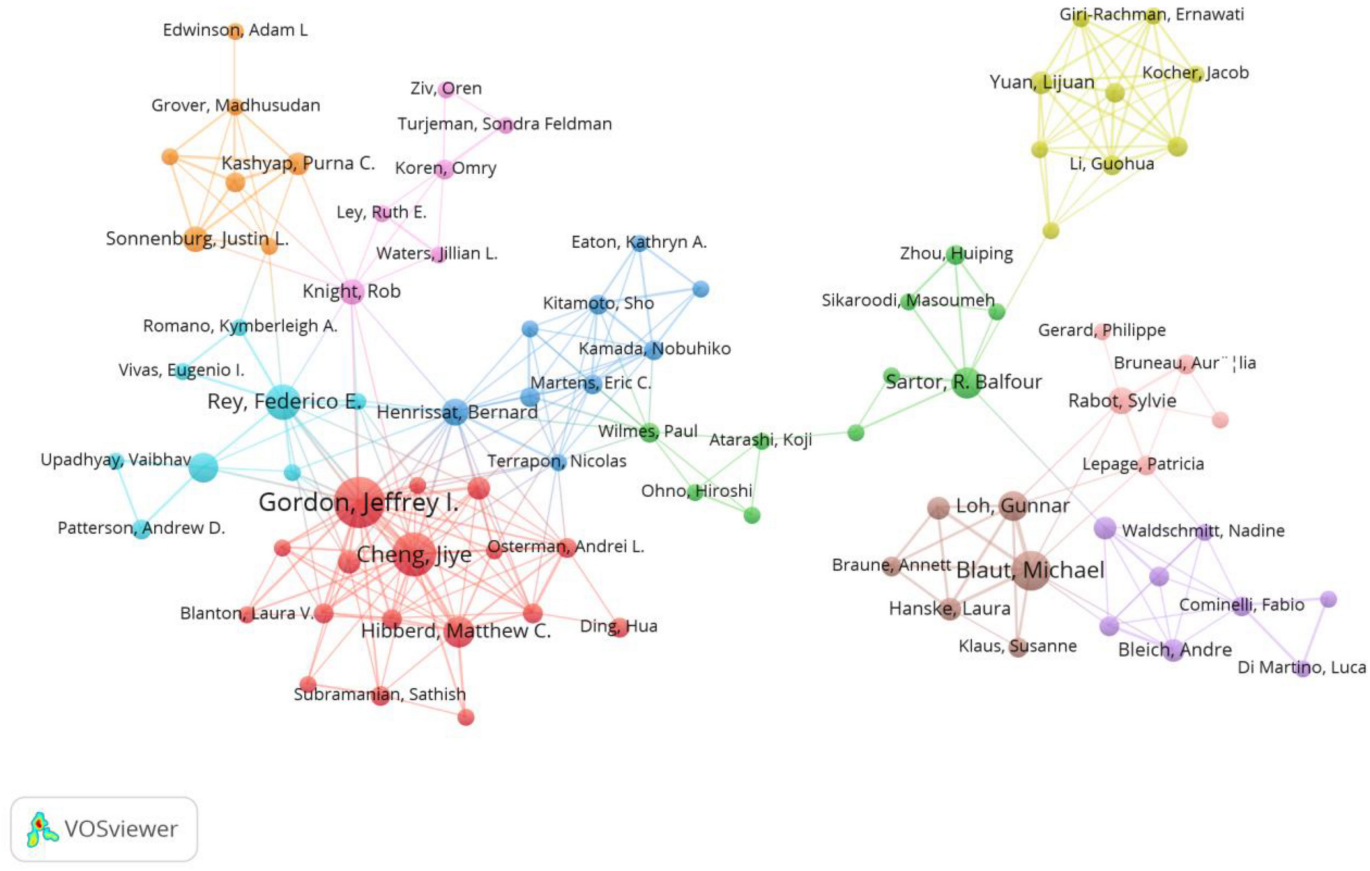
Visualization of authors’ distribution and collaboration. Each node in the figure represents an author, with node size proportional to their publication count. The thickness of the connecting lines reflects collaboration frequency. Nodes of different colors represent distinct collaboration networks.

Figure 5 displays the VOSviewer generated visualization of author collaboration networks. As shown in Figure 5, ten collaboration networks exist worldwide, each comprising more than five authors. Specifically, Jeffrey I. Gordon and Cheng Jiye, both affiliated with Washington University, serve as central nodes. They co-author nine publications and maintain close collaborations with many other researchers, indicating their core status in the field. In addition, some isolated nodes are visible, reflecting limited collaboration networks for those authors who may be early-career researchers or scholars focusing on specific niche topics. For example, Yuan Lijuan and Li Guohua mainly conduct research on “human microbiota-gnotobiotic pig model-immunity.” Overall, the figure provides researchers with a clear perspective for understanding the collaboration patterns among authors and their positions within the scholarly network.

### 3.6 Journal distribution

For this topic, 231 distinct journals worldwide have published relevant articles. Table 4 lists the ten most productive journals together with their article counts, ACI, H-index, impact factor (IF), and Journal Citation Reports (JCR). Nine of them belong to the JCR Q1 quartile, whereas PLOS One falls into JCR Q2. The highest-ranking journals in terms of ACI and IF are *Science* (ACI = 1728.00), *Nature* (ACI = 1425.92), and *Cell* (ACI = 819.33). Moreover, the leading journals by publication volume and H-index are *Gut Microbes* (*N* = 32, H-index = 17), *Microbiome* (*N* = 22, H-index = 17), and *Proceedings of the National Academy of Sciences of the United States of America (PNAS)* (*N* = 18, H-index = 15).

**TABLE 4:** Top 10 journals.

Rank	Journal	Quantity	ACI	H-index	IF (2024)	JCR
1	Gut Microbes	32	33.78	17	11	Q1
2	Microbiome	22	125.59	17	12.7	Q1
3	Proceedings of the National Academy of Sciences of the United States of America	18	374.00	15	9.4	Q1
4	Cell	15	819.33	15	45.5	Q1
5	Gut	14	154.64	13	14.69	Q1
6	Frontiers In Microbiology	14	29.29	11	5.9	Q1
7	Nature	12	1425.92	12	69.5	Q1
8	PLOS One	11	64.45	11	3.2	Q2
9	Science	11	1728.00	11	63.7	Q1
10	Frontiers In Immunology	10	28.90	10	7.1	Q1

ACI, average citations per item; IF, impact factor; JCR, journal citation reports.

**FIGURE 6:**
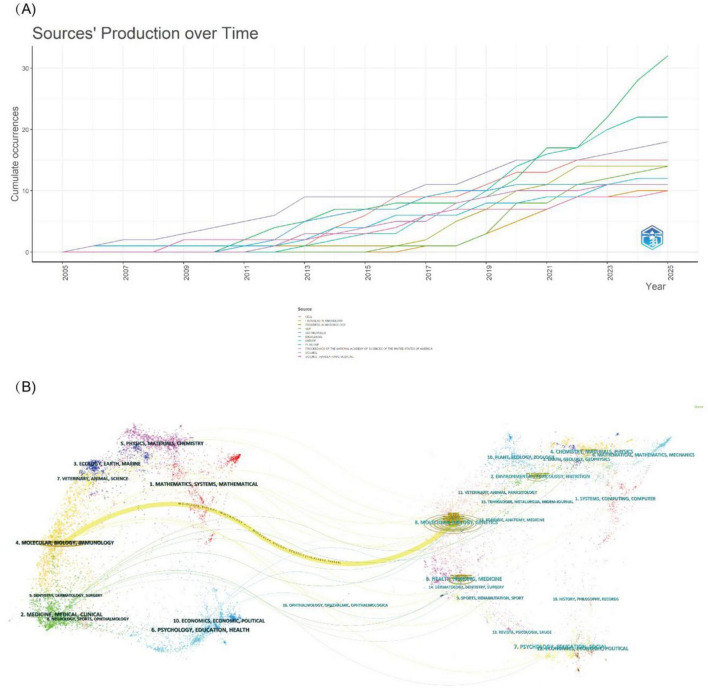
Visualization of Journal publication trends and research landscape. **(A)** Temporal trend of annual publication output for the top 10 journals ranked by cumulative publications from 2005 to 2025. **(B)** The dual-map overlay of journal publishing research. Cited journals are shown in black, and citing journals are in blue. Ellipses represent the number of authors and publication volume for specific journals.

Figure 6A, produced with Bibliometrix, depicts the distribution and temporal evolution of journal output. *PNAS* had maintained the highest number of publications up to 2020, but since 2021, it has been overtaken by *Gut Microbes* and *Microbiome*. Since its launch in 2010, *Gut Microbes* has shown a steady increase in topic-related papers, culminating in a dramatic surge in 2022 that elevated it to the leading position in terms of article count. The citation and co-citation relationships among journals were visualized using VOSviewer. As shown in Figure 6B, the principal citing journals cluster in *Molecular Biology, Immunology, Medicine*, and *Medical and Clinical Studies*, whereas the most frequently cited journals are concentrated in *Health, Nursing, Medicine, Molecular Biology, and Genetics*.

### 3.7 Research hotspots and frontiers

Table 5 lists the ten keywords with the highest occurrence frequencies. The six leading keywords: “intestinal microbiota,” “mice,” “humans,” “animal model,” “animals,” and “nonhuman”—serve as subject headings or free-text terms describing humans, microbiota, and animal or non-human model systems. Additional terms, for example, “controlled study” and “microbiology,” indicate the research design and associated disciplinary domains.

**TABLE 5:** Top 10 keywords.

Rank	Keywords	Count	Total link strength
1	Intestinal microbiota	451	7324
2	Mice	231	5845
3	Humans	218	6122
4	Animal model	201	5380
5	Animals	199	5846
6	Nonhuman	196	5834
7	Article	194	5829
8	Controlled study	158	4973
9	Microbiology	155	4507
10	Metabolism	132	3027

**FIGURE 7:**
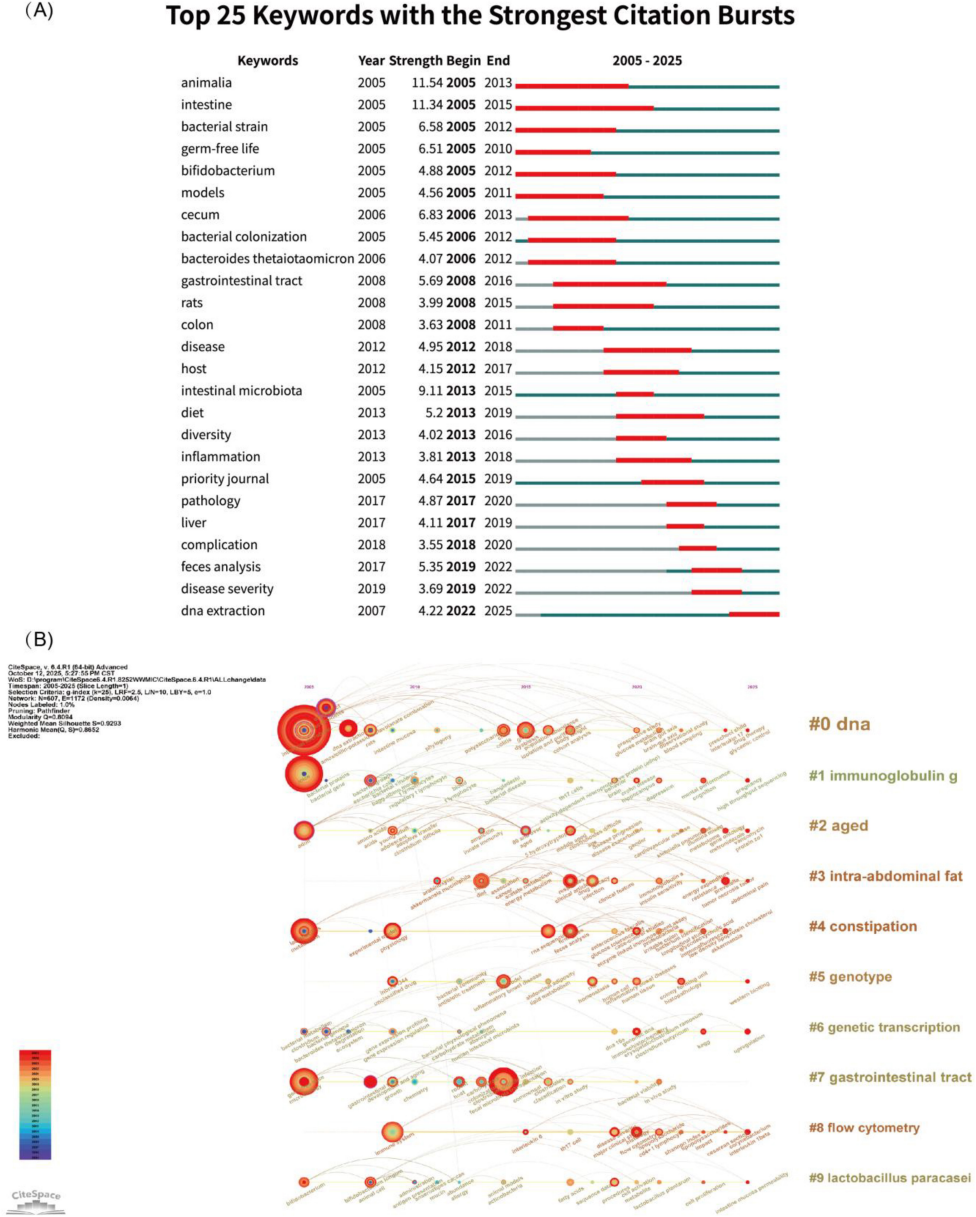
Visualization of keyword bursts and timeline clustering analysis. **(A)** Top 20 keyword bursts analysis. The green horizontal axis represents the timeline of keyword appearance, and the red bars indicate the duration of keyword bursts. **(B)** Keyword timeline clustering analysis. The horizontal axis in the figure represents the timeline, with labeled horizontal lines on the right indicating the clustering of keywords. The nodes on the horizontal lines represent individual keywords, with their positions indicating the year of first appearance in the relevant literature. The size and color intensity of the nodes reflect the frequency and duration of the keywords, respectively. Keywords with a higher number of occurrences in the cluster are ranked higher.

Keyword bursts were detected with CiteSpace to review and forecast the stage-wise hotspots and developmental trends of this field (Figure 7A). In 2005, several keywords emerged and burst, including “animalia,” “intestine,” “bacterial strain,” “germ-free life,” “bifidobacterium,” and “models.” “Animalia” (burst strength = 11.54) and “intestine” (burst strength = 11.34) displayed the highest intensities, reflecting their prominent focus in the initial research phase. “Intestine” also maintained the longest burst duration, persisting for 10 years, whereas “animalia” and “gastrointestinal tract” each sustained bursts for 8 years. These keywords, characterized by strong bursts and extended relevance, indicate that studies within the HMA animal models domain focus chiefly on allogeneic gut-microbiota transfer and probiotic regulation, like *Bifidobacterium*. Since 2022, the only keyword whose burst continues to the present is “DNA extraction,” implying that novel DNA-extraction and detection technologies may become the near-term and future focus of this topic.

CiteSpace also visualized keyword clustering and its chronological evolution (Figure 7B). “DNA” is the earliest, most extensive, and longest-persisting keyword cluster, reflecting the sustained interest in the advancement of DNA-extraction and assay technologies within microbial studies. Later, terms like “immunoglobulin G” and “aged” became focal keywords, highlighting the growing attention to the interplay among human immune function, aging, and the microbiota. Recently, the increasing number of nodes associated with “intra-abdominal fat,” “constipation,” and “flow cytometry” signals their emergence as new research foci, pointing to future directions for the field.

### 3.8 Reference citation and bursts

Table 6 lists a compilation of the ten publications with the highest citation counts. The top-ranked article is the 2006 *Nature* paper by Gordon et al. (2006), “*An obesity-associated gut microbiome with increased capacity for energy harvest*,” which has received 10,240 citations. This paper initially demonstrated that transferring the gut microbiota of obese donors into GF mice increases the mice’s dietary energy extraction capacity, thereby recapitulating the obesity phenotype. These findings indicate that transplantation of human gut microbiota into animals can partially reproduce disease phenotypes and facilitate mechanistic investigations. Consequently, HMA animal models have garnered widespread interest, prompting numerous related studies. The works ranked 2nd, 3rd, 5th, and 9th similarly focus on the bidirectional interactions among cancer, immunotherapy, and the intestinal microbiome. The remaining top-cited articles examine the associations of obesity, Parkinson’s disease (PD), and regulatory T (Treg) cells with the human microbiota. Moreover, two studies highlight how diet and host genetics influence human-microbiota transplantation outcomes and the consequent compositional differences. These observations imply that metabolic conditions (e.g., obesity), neurodegenerative disorders (e.g., PD), immune-related diseases, together with dietary and genetic factors affecting the microbiome, have attracted considerable attention in this research area.

**TABLE 6:** Top 10 highly cited publications.

Rank	Title	Citations	Year	DOI
1	An obesity-associated gut microbiome with increased capacity for energy harvest	10,240	2006	10.1038/nature05414
2	Gut microbiome influences efficacy of PD-1-based immunotherapy against epithelial tumors	4,348	2018	10.1126/science.aan3706
3	Gut microbiome modulates response to anti-PD-1 immunotherapy in melanoma patients	3,439	2018	10.1126/science.aan4236
4	Gut microbiota from twins discordant for obesity modulate metabolism in mice	3,097	2013	10.1126/science.1241214
5	Anticancer immunotherapy by CTLA-4 blockade relies on the gut microbiota	2,900	2015	10.1126/science.aad1329
6	Gut Microbiota Regulate Motor Deficits and Neuroinflammation in a Model of Parkinson’s Disease	2,572	2016	10.1016/j.cell.2016.11.018
7	The effect of diet on the human gut microbiome: A metagenomic analysis in humanized gnotobiotic mice	2,463	2009	10.1126/scitranslmed.3000322
8	Human Genetics Shape the Gut Microbiome	2,236	2014	10.1016/j.cell.2014.09.053
9	The commensal microbiome is associated with anti-PD-1 efficacy in metastatic melanoma patients	2,230	2018	10.1126/science.aao3290
10	Treg induction by a rationally selected mixture of Clostridia strains from the human microbiota	2,209	2013	10.1038/nature12331

DOI, digital object identifier.

Table 7 lists the ten references with the highest co-citation numbers. The top-ranked article is the 2009 *Science Translational Medicine* paper by Turnbaugh et al. (2009), “*The effect of diet on the human gut microbiome: a metagenomic analysis in humanized gnotobiotic mice*,” which has received 69 co-citations. The second-ranked reference is the 2013 *Science* article by the same senior author, Ridaura et al. (2013), “*Gut microbiota from twins discordant for obesity modulate metabolism in mice*,” which has received 59 co-citations. Together, these two studies confirm that HMA mouse models can transfer disease phenotypes to the host, thereby establishing the basis for research on HMA animal models. Subsequently, the 2010 *Nature Methods* paper by Caporaso et al. (2010), “*QIIME allows analysis of high-throughput community sequencing data*,” which has received 50 co-citations, introduced a widely adopted analytical pipeline for microbial community analysis. Of the remaining seven references, one introduces a new methodological approach for microbial analysis; another provides a metagenomic survey of the human intestinal microbiota; a third investigates dietary influences on the human gut microbial community. Finally, the remaining four studies focus on the interplay among immune function, lipid metabolism, and the intestinal microbiome. Collectively, these findings demonstrate that research on HMA animal models primarily focuses on advancing analytical technologies, refining knowledge of human microbial composition, elucidating determinants of humanized animal-model construction, and validating disease mechanisms.

**TABLE 7:** Top 10 highly co-cited references.

Rank	Title	Co-citations	Year	DOI
1	The effect of diet on the human gut microbiome: a metagenomic analysis in humanized gnotobiotic mice	69	2009	10.1126/scitranslmed.3000322
2	Gut microbiota from twins discordant for obesity modulate metabolism in mice	59	2013	10.1126/science.1241214
3	QIIME allows analysis of high-throughput community sequencing data	50	2010	10.1038/nmeth.f.303
4	An obesity-associated gut microbiome with increased capacity for energy harvest	49	2006	10.1038/nature05414
5	DADA2: High-resolution sample inference from Illumina amplicon data	42	2016	10.1038/nmeth.3869 10.1038/nmeth.3869
6	A human gut microbial gene catalog established by metagenomic sequencing	41	2010	10.1038/nature08821
7	Gut immune maturation depends on colonization with a host-specific microbiota	35	2012	10.1016/j.cell.2012.04.037
8	A core gut microbiome in obese and lean twins	35	2009	10.1038/nature07540
9	Diet rapidly and reproducibly alters the human gut microbiome	32	2014	10.1038/nature12820
10	The gut microbiota as an environmental factor that regulates fat storage	30	2004	10.1073/pnas.0407076101

DOI, digital object identifier.

**FIGURE 8:**
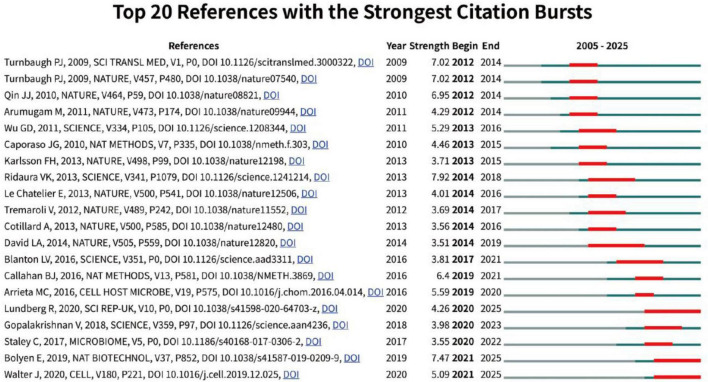
Citation-burst analysis of references. Burst patterns of cited references visualized by CiteSpace, highlighting the 20 references that exhibited the strongest citation surges.

Furthermore, CiteSpace was employed to visualize the burst patterns of the cited references (Figure 8). The most intense citation burst is observed by Ridaura et al. (2013), titled “*Gut microbiota from twins discordant for obesity modulate metabolism in mice*,” indicating that the interplay among diet, gut microbiota, and metabolism attracted the greatest attention from 2014 to 2018. Currently, three papers still show citation bursts: (1) Bolyen et al. (2019) publication “*Reproducible, interactive, scalable and extensible microbiome data science using QIIME 2*”; (2) Lundberg et al. (2020) study “*Human microbiota-transplanted C57BL/6 mice and offspring display reduced establishment of key bacteria and reduced immune stimulation compared to mouse microbiota-transplantation*”; and (3) Walter et al. (2020) article “*Establishing or Exaggerating Causality for the Gut Microbiome: Lessons from Human Microbiota-Associated Rodents*.” Collectively, these studies address distinct topics. They illustrate that, in the field of HMA animal models, investigators focus on emerging analytical methods, microbial-immune interactions, and the utility of the allogeneic transplant animal models for establishing causal relationships between the microbiota and disease.

## 4 Discussion

### 4.1 General information

In this research, we applied bibliometric techniques, using specialized tools including VOSviewer, CiteSpace, and Bibliometrix to thoroughly examine publications concerning human microbiota-associated (HMA) animal models research from 2005 through 2025. Our findings show that the annual output of relevant papers has continuously increased over the past two decades, reflecting a sustained worldwide scholarly interest in HMA animal model studies. The analysis of contributions by country, region, and institution demonstrates that the USA and its affiliated institutions produce the highest number of publications, whereas France, Sweden, and Denmark achieve the highest mean citations per paper, highlighting the dominant position and extensive influence of Western nations. Moreover, China exhibits strong expansion in this field, retaining the second-largest worldwide output and fostering active cooperation with Western nations.

Regarding authorship distribution and collaboration, Jeffrey I. Gordon of Washington University has the highest number of publications. The research of Jeffrey I. Gordon focuses on “*gut microbiome, nutrition, and energy metabolism*” (Turnbaugh et al., 2006, 2009; Ridaura et al., 2013). Moreover, Jeffrey I. Gordon and Cheng Jiye jointly investigated “*gut microbiota, development, and nutrition*,” producing multiple highly-cited articles (Hibberd et al., 2017; Cowardin et al., 2019; Coskun et al., 2025). The aforementioned researchers primarily transplant human microbiota into germ-free (GF) mice to assess how the transplanted microbiota influences disease processes. In contrast, Yuan Lijuan and Li Guohua of Virginia Polytechnic Institute and State University established a gnotobiotic (Gn) pig model using fecal microbiota transplantation (FMT) (Zhang et al., 2014; Twitchell et al., 2016; Wang et al., 2016). Relative to the standard GF mouse model, GF pigs exhibit human-like physiology, metabolism, biochemistry, and genetics, making pig models of the human microbiome equally valuable (Wang and Donovan, 2015). Regarding journal distribution, *Gut Microbes* and *Microbiome* serve as the leading outlets for studies on gut microbiota and broader microbial science. Since their launch, the total number of articles has continuously increased, with a marked acceleration after 2018, which reflects an escalating focus on HMA animal models.

Regarding research hotspots and frontiers, the most intense and persistent burst keywords include “animalia,” “intestine,” “gastrointestinal tract,” “immunoglobulin G,” “aged,” “intra-abdominal fat,” and “constipation.” This suggests that within HMA animal models, the gut microbiome’s links to immunity, senescence, adiposity, and constipation have not only been historical research foci but also represent crucial avenues for future investigation. Specifically, intra-abdominal fat and constipation correspond to metabolic disease (obesity) and digestive system disease, respectively, implying that the gut microbiota holds sustained research prospects in both disease categories. Regarding reference citations and burst patterns, the most frequently cited and co-cited works concentrate on the gut microbiome, metabolic disorders (obesity), cancer, neurodegenerative diseases (Parkinson’s disease), immune function, nutrition, and genetics, underscoring the pivotal role of HMA animal models in these fields. Subsequently, we will conduct a thorough investigation of how these diseases interact with HMA animal models and examine the impacts of dietary, genetic, and other variables on such models.

### 4.2 Hotspots and frontiers

#### 4.2.1 Metabolic disease and HMA animal models

Earlier research indicates that the intestinal microbiome is intimately connected with human host metabolism, and disturbances in this microbiome are related to the development of metabolic disorders, including obesity, diabetes, non-alcoholic fatty liver disease, and malnutrition (Fan and Pedersen, 2021). When exploring associations between intestinal microbes and disease, HMA animal models constitute the most effective means of validation. For metabolic disorders, the conventional methodologies employing HMA animal models can be divided into three categories:

First, researchers generate HMA models by FMT to evaluate the extent to which the gut microbiome can affect host metabolic processes. For example, Ridaura et al. (2013) reported that GF C57BL/6J mice colonized with microbiota from obese twins showed markedly increased body weight and adiposity relative to mice colonized with microbiota from lean twins. Wang et al. (2025) transferred fecal microbiota from individuals with type-1 diabetes into GF mice, which subsequently displayed a phenotype of disrupted glucose metabolism phenotype. Wang et al. (2020) introduced fecal microbiota from end-stage renal disease (ESRD) patients into GF mice with kidney injury or into rats treated with antibiotics. This resulted in elevated serum uremic toxins, intensified renal fibrosis and oxidative stress, and pinpointed *Eggerthella lenta* and *Fusobacterium nucleatum* as pivotal microbes driving disease advancement (Wang et al., 2020). In another study, Wang et al. (2018) transferred gut microbiota collected from genetically obese individuals before and after dieting into GF male C57BL/6J mice. The pre-diet microbiota modulated hepatic gene expression related to lipid metabolism, induced macrovesicular steatosis, and raised liver triglyceride and cholesterol concentrations, suggesting a possible pathogenic pathway for non-alcoholic steatohepatitis (NASH) (Wang et al., 2018).

Second, the creation of HMA models permits investigation of the therapeutic mechanisms arising from the interplay between probiotics or pharmacological agents and the intestinal microbiome. For instance, administration of *Bifidobacterium animalis* to HMA-ESRD mice ameliorated the abundance of pathogenic microbes, lowered circulating toxin levels, and slowed disease progression (Wang et al., 2020). Using fecal microbiota from NASH patients combined with a Western diet, an HMA-NASH mouse model was established; subsequent treatment with Colesevelam alleviated body-weight gain, hepatic inflammation, steatosis, and insulin resistance (Hartmann et al., 2022).

Third, by generating HMA animals colonized with microbiota from healthy donors and then applying disease-inducing experimental manipulations, researchers can evaluate the contribution of the intestinal microbiome to disease development. As an illustration, Vlasova et al. (2017) introduced fecal microbiota from healthy newborns into GF piglets and provided either protein-deficient milk feedings, establishing an HMA model of childhood malnutrition in neonatal pigs that mirrors the compromised immune function, gut integrity, and other physiological deficits seen in malnourished children (Miyazaki et al., 2018; Vlasova et al., 2017). Within the pediatric malnutrition pig model, subsequent investigations of human rotavirus infection and vaccine performance demonstrated that heightened susceptibility and diminished vaccine efficacy were associated with microbiome changes induced by malnutrition (Kumar et al., 2018; Srivastava et al., 2020) (Supplementary Table 1).

In summary, intestinal microbiota are integral to the onset and progression of metabolic disorders. Employing HMA animal models enables investigators to dissect the influence of microbial communities on host metabolism and to identify prospective therapeutic approaches, thereby providing critical experimental foundations for understanding disease pathogenesis and for designing novel therapeutic modalities.

#### 4.2.2 Digestive system disease and HMA animal models

According to estimates, over 95% of the microbial community in the human body is located in the gut (Sender et al., 2016). To elucidate the functional mechanisms of the intestinal microbiome, investigators have utilized FMT to establish HMA gastrointestinal models in pigs (Zhang et al., 2013; Rose et al., 2022) and mice (Chung et al., 2012; Yang et al., 2025). Based on these HMA animal models, tight associations have been uncovered between the gut microbiome and essential physiological functions, including intestinal development (Yu et al., 2016), peristalsis (Dey et al., 2015), and immune responses (Chung et al., 2012). In particular, various microbial consortia affect intestinal architecture—altering villus length, crypt depth, epithelial proliferation, and the populations of goblet and Paneth cells—which in turn governs epithelial cell differentiation and maturation along the gut (Yu et al., 2016). Secondly, gut motility is largely dictated by dietary inputs and the composition of the microbial community. Research involving dietary manipulation of HMA mice indicates that nutritional regimens affect intestinal motility by modulating the vitality, composition, and migration of the gut microbiota (Kashyap et al., 2013; Dey et al., 2015) A third point is that intestinal immune maturation is heavily reliant on co-evolved, niche-specific microbes, which trigger activation and proliferation of T cells in gut lymphoid structures, driving T-cell amplification and the refinement of mucosal immunity (Chung et al., 2012). Because intestinal physiological processes are tightly coupled with the microbiome, dysbiosis plays a pivotal role in the pathogenesis and evolution of gut disorders. In a study by Gray et al. (2024), fecal microbiota from inflammatory bowel disease patients were introduced into GF mice. The resulting host inflammation fostered the overgrowth of invasive commensals, worsening gut inflammation and causing dysbiosis that further intensified inflammation, establishing a vicious circle that propels disease advancement (Gray et al., 2024). Using HMA mouse models, Chen et al. (2018) examined the link between the intestinal microbiome and the etiology and development of irritable bowel syndrome, pinpointing *Parasutterella* as a likely pivotal species. By integrating HMA with ATG16L1 T300A knockout mice, Lavoie et al. (2019) confirmed that this Crohn’s disease susceptibility allele triggers microbial imbalance and immune cell infiltration, hastening disease development. In addition, there is a reciprocal relationship between bacterial pathogens and the intestinal microbial community. Using HMA animal models, Bratburd et al. (2018) performed infection experiments with the pathogenic *Salmonella enterica* or the opportunistic *Candida albicans*, revealing substantial shifts in the intestinal microbiome and its metabolite profile, especially after *S. enterica* challenge. The increased metabolites are likely to be pivotal in the oxidative stress triggered by these pathogens (Bratburd et al., 2018) (Supplementary Table 1).

Overall, the intestinal microbiome occupies a core role in sustaining gut physiology, influencing disease pathogenesis, and modulating the interplay with bacterial pathogens. Subsequent research ought to dissect the intricate mechanisms of the intestinal microbiome, thereby providing solid scientific rationale and novel approaches for accurate diagnosis, therapeutic intervention, and prophylaxis of gut disorders.

#### 4.2.3 Cancer and HMA animal models

In recent years, a growing body of evidence has demonstrated that the intestinal microbiome contributes to tumor development and immunotherapy (Xie and Liu, 2024). Using HMA animal models, investigators have extensively examined how the intestinal microbial community interacts with cancers, including colorectal cancer (CRC), melanoma, and pancreatic adenocarcinoma.

In CRC research, Baxter et al. (2014) transferred fecal microbiota from CRC patients and healthy donors into GF mice, which subsequently induced tumors. They reported a positive association between the abundance of *Bacteroides, Parabacteroides, Alistipes*, and *Akkermansia* with tumor load, suggesting that the structure of the gut microbiome is tightly associated with the likelihood of colorectal tumor formation (Baxter et al., 2014). Sui et al. (2020) transferred fecal material from participants who received the Chinese herbal decoction Yi-Yi-Fu-Zi-Bai-Jiang-San (YYFZBJS) into C57BL/6J Apc^Min/+^GF mice, a model of intestinal tumorigenesis. The intervention promoted T-cell activation and consequently inhibited the proliferation of CRC cells. Parker et al. (2021) introduced fecal microbiota from CRC survivors with a long-term rice-bran diet into GF mice. This resulted in a significant enrichment of health-promoting taxa (e.g., *Flavonifractor, Oscillibacter*), elevated levels of anticancer metabolites, and a consequent decrease in chemically induced tumor incidence (Parker et al., 2021). In melanoma research, Gopalakrishnan and Matson revealed that differences in patient outcomes to anti-programmed cell death 1 protein (PD-1) immunotherapy could be microbiota-dependent. When fecal microbiota from the responders were transferred to GF melanoma-bearing mice, T-cell activity was amplified, and the therapeutic benefit of anti-PD-1 treatment was markedly increased (Gopalakrishnan et al., 2018; Matson et al., 2018). Additionally, Teng et al. (2025) treated melanoma HMA mice with ginger-originated plant extracellular vesicles. They demonstrated that miR-159a-3p reshaped the gut microbiome’s metabolic profile, boosted docosahexaenoic acid (DHA) synthesis, down-regulated tumor programmed death-ligand 1 (PD-L1), and substantially improved the response to anti-PD-L1 immunotherapy (Teng et al., 2025). In pancreatic cancer research, Genton et al. (2021) reported that transplantation of pancreatic-cancer patient microbiota into GF mice led to a significant reduction in visceral fat mass in the recipients. Tintelnot et al. (2023) found that, within HMA mice models of pancreatic ductal adenocarcinoma, FMT as well as dietary tryptophan or its downstream metabolite indole-3-acetic acid (3-IAA) significantly improved chemotherapeutic outcomes (Supplementary Table 1).

Overall, evidence from HMA animal models investigations underscores the intricate and essential contributions of the intestinal microbiome to tumorigenesis, disease progression, and immunotherapeutic responsiveness in colorectal cancer, melanoma, and pancreatic cancer. Employing approaches like FMT enables the pinpointing of tumor-relevant microbial signatures and clarifies the mechanisms by which the microbiome influences host immunity, metabolite profiles, and therapeutic drug performance. Such insights advance our comprehension of gut-tumor interactions and provide a solid basis for the creation of microbiome-targeted cancer diagnostics and treatment modalities.

#### 4.2.4 Nervous system disease and HMA animal models

The notion of a “gut-brain axis” underscores a two-way communication between the gut and the central nervous system (Dinan and Cryan, 2017), but the underlying molecular pathways have not been fully clarified. HMA animal models serve as a crucial link between fundamental studies and clinical practice (Socała et al., 2021), furnishing methodological foundations to investigate how intestinal microbiota influence the gut-brain axis and neurological diseases. Earlier studies have shown that, during the perinatal period, the intestinal microbial community and the nervous system undergo concurrent early developmental phases (Lu and Claud, 2019). It has been reported that transferring the microbiota of preterm infants into GF mice modifies microbial succession and metabolite production, which in turn impairs adult associative memory and learning (Lu et al., 2022). In a comparable experiment, Lu et al. (2018) introduced the microbiota from growth-impaired preterm infants into pregnant GF mice, resulting in offspring with postponed neuronal and oligodendrocyte development as well as reduced myelin formation. Moreover, Lu et al. (2023) transplanted necrotizing enterocolitis-derived fecal microbiota into pregnant GF mice and found that the offspring displayed gut inflammation, postponed neuronal maturation, diminished oligodendrocyte myelination, white-matter damage, and impaired motor and cognitive functions (Supplementary Table 1). Collectively, these findings indicate that an altered intestinal microbiome in preterm infants exerts adverse and lasting effects on cerebral maturation and neural function.

Beyond these contexts, intestinal microbes also appear to play a pivotal role in various other neuro-diseases. For example, Sampson et al. (2016) introduced microbiota from PD patients into GF mice engineered to overproduce α-synuclein, which led to a pronounced worsening of motor impairment, implying that gut microbes could be critical facilitators of α-synuclein-driven PD (Supplementary Table 1). In another study, Berer et al. (2017) colonized GF mice susceptible to spontaneous autoimmune encephalomyelitis with microbiota from multiple sclerosis patients, observing a marked increase in disease onset, which highlights a potential pathogenic contribution of an altered gut microbiome to neuroimmune diseases.

Overall, HMA animal models have demonstrated both the pivotal influence of intestinal microbiota on the cerebral maturation of preterm infants and the possible disease-causing pathways in multiple neuro-conditions. Together, the findings enrich the empirical support for microbiome-based therapeutic strategies for neurological disorders.

#### 4.2.5 Influence factors of “human microbiota-animal models”

The establishment of HMA animal models through FMT requires four main procedures: (1) choosing appropriate donors based on predefined inclusion and exclusion criteria; (2) gathering donor stool and preparing a fecal microbiota suspension; (3) delivering the suspension to the animals via gavage or enema; (4) assessing colonization efficiency by culture or high-throughput sequencing (Wang and Donovan, 2015; Gheorghe et al., 2021; Ruszkowski et al., 2025). Microbial colonization efficiency in the recipient animal is mainly affected by the type of animal, age, and the feeding diet (Park and Im, 2020; Huang et al., 2025). Current evidence indicates that, apart from the host’s genetic background, diet is the most prominent variable influencing HMA animal models.

Diet serves as the primary determinant of microbial community structure; various dietary sources (animal, plant, or combined) can swiftly reshape microbial abundance and functional profiles, influencing host wellbeing (Zmora et al., 2019). In standard laboratory mice, research has shown that identical genetic backgrounds subjected to distinct dietary regimes develop significant microbiota disparities (Zmora et al., 2019). Nevertheless, given that about 40% of the microbial community is transmissible across generations, genotypes of different mouse strains can partially regulate the strength of diet-induced changes (Huda et al., 2022). Consequently, the design of HMA animal models should account for both diet and host genetics. For example, using a humanized diet in HMA mice raises colonization rates by 10% (Moreno-Indias et al., 2020). The microbial diversity of BALB/c mice is typically greater than that of other strains, notably in immunocompromised models such as non-obese diabetic or severe combined immunodeficient mice (Park and Im, 2020). Conversely, C57BL/6J mice display a heightened sensitivity to gut microbiota changes relative to other mouse strains (Huda et al., 2022). If required, specific control groups can clarify the relative contributions of diet versus genetics to disease phenotypes. Wang and colleagues, for instance, contrasted high-fat, high-sugar, and regular chow in leptin-deficient (ob/ob) mice and wild-type controls, observing that diet exerted a stronger impact on the gut microbiome than genotype, underscoring diet as the leading factor in obesity studies (Wang et al., 2019). In a study by Eun and co-workers, different strain-specific knockout mice colonized with human microbiota revealed that host genetics modulates colonization efficiency. The 129S6/SvEv mice were more susceptible than the C57BL/6, provoking stronger immune activation and colitis (Eun et al., 2014).

In summary, the construction of HMA animal models should employ a diet that replicates human eating habits, thereby enhancing colonization efficiency. Moreover, choose mouse strains that exhibit pronounced diet-microbiota interplay or that readily accept human microbial communities for control studies. If feasible, implement several diet regimens within a uniform genetic background and several strains under the same diet to separate the distinct effects of nutrition and host genotype. Considering diet and host genetic variables in an integrated manner enables improved colonization success and a more robust interpretation of disease pathways in these models, thereby furnishing a solid foundation for future translational studies.

### 4.3 Advantages and limitations

This study applied bibliometric techniques to conduct a systematic search, statistical aggregation, and visual mapping of scholarly publications on HMA animal models. The objective was to delineate research trends, collaborative networks, and emerging hotspots within the field. Inevitably, several limitations exist: (1) the query terms were predominantly English, thereby omitting non-English literature; (2) cumulative citation counts and the H-index evolve over time, so the present metrics only capture historical influence and cannot reflect the most recent research impact in real time; (3) bibliometric analysis focuses on quantity and structure, making it difficult to assess the experimental quality and innovativeness of individual studies, which may underestimate high-quality work with low output. In spite of these limitations, the present study provides a comprehensive quantitative overview of HMA animal models research, providing meaningful insights into both ongoing and forthcoming related studies.

## 5 Conclusion

This bibliometric analysis was conducted on publications related to HMA animal models from 2005 to 2025, offering a comprehensive overview of the field’s production trends, academic landscape, and emerging research directions. The continuous increase in the volume of publications from 2005 to 2025 reflects the growing global interest in intestinal microbes and their respective animal models. The USA and its institutions were the largest contributors; although France, Sweden, Denmark and similar nations generated fewer outputs, they recorded the highest mean citations per paper, underscoring their dominance in high-impact studies. The analysis of keywords and top-cited papers indicates that areas including intestinal microbiota, senescence, metabolic processes, oncology, neurodegeneration, immune function, nutrition, and host genetics have persistently attracted intense and long-term scholarly attention. Going forward, HMA animal models will remain essential connectors of clinical and fundamental microbiome research. Accordingly, the community should agree on unified, standardized, and workflow-driven preparation protocols and should implement quality-assurance criteria to enhance model repeatability and the reliability of investigations into disease causality.

## Data Availability

The original contributions presented in the study are included in the article/Supplementary material, further inquiries can be directed to the corresponding author.
