# Supplementary material for: Bibliometric analysis of human microbiota-associated animal model (2005–2025)
Source: Front Microbiol. 2026 Apr 30;17:1777297. doi: 10.3389/fmicb.2026.1777297 (PMC13171774; doi:10.3389/fmicb.2026.1777297)
Supplement: Supplementary file 1 [file Table_1.docx]

**Supplementary Table 1** Overview of human microbiota-associated animal models in diseases

| Disease | Model | Colonization/Experiment | Human disease recapitulation & outcome | Reference | DOI |
| --- | --- | --- | --- | --- | --- |
| Obesity | GF male C57BL/6J mice | Donor: 4 pairs of twins with differing obesity status, as well as from cultured microbes originating from either an obese or lean co-twin.  Methodology: single oral gavage. Mice were fed a chow diet or one of two diets (high or low in saturated fats) | The obesity and metabolic phenotype were transferable to humanized mice, and co-housing of obese and lean mice mitigated the phenotype. | (Ridaura et al., 2013) | 10.1126/science.1241214 |
| Type 1 diabetes | GF male C57BL/6 mice | Donor: 5 patients with type 1 diabetes and 4 healthy people. Methodology: oral gavage, once a day for three times. | The microbiota from type 1 diabetes patients successfully reproduced the donors’ glucose‑metabolism disorder in recipient mice: elevated blood glucose, increased OGTT AUC, and abnormal insulin levels. Phenotype reproducibility was tightly linked to microbiota compositional similarity. | (Wang et al., 2025) | 10.1007/s11427-024-2658-1 |
| End-stage renal disease | GF C57BL/6 mice or antibiotic-treated SD rats | Donor:13 end-stage renal disease patients.  Methodology: oral gavage over the first week. Mice were fed with either 0.2% adenine-containing diet. Rats underwent the 5/6 nephrectomy surgery. | Microbiota from patients transplanted to renal-injured GF mice or antibiotic-treated rats induce higher production of serum uraemic toxins and aggravated renal fibrosis and oxidative stress. | (Wang et al., 2020) | 10.1136/gutjnl-2019-319766 |
| Non-alcoholic steatohepatitis | GF male C57BL/6J mice | Donor: a patient with Prader-Willi syndrome  Methodology: oral gavage for 2 consecutive days. Mice were fed a sterile normal chow diet. | Liver macrovesicular steatosis, together with elevated hepatic triglycerides and cholesterol levels, was transmissible to humanized mice. | (Wang et al., 2018) | 10.3389/fmicb.2018.01602 |
| Non-alcoholic steatohepatitis | GF male C57BL/6 mice | Donor: 2 patients with non-alcoholic steatohepatitis.  Methodology: oral gavage, repeated 2 weeks later. Mice were subjected to 20 weeks of Western diet feeding with and without colesevelam. | Colesevelam attenuated western‑diet‑induced body and liver weight gain, reduced hepatic inflammation, steatosis, fibrosis and insulin resistance, and promoted de novo bile‑acid synthesis while lowering hepatic cholesterol in microbiome‑humanized mice. | (Hartmann et al., 2022) | 10.1007/s12072-022-10296-w |
| Malnourished children | GF pig | Donor: a healthy, 2-month-old, exclusively breastfed infant.  Methodology: oral gavage. Pigs were fed protein-deficient or sufficient bovine milk diets. | Protein deficiency induced hypoproteinemia, hypoalbuminemia, hypoglycemia, stunting, and generalized edema in gnotobiotic pigs, mirroring the condition observed in protein‑malnourished children. | (Miyazaki et al., 2018; Vlasova et al., 2017) | 10.1016/j.vaccine.2018.09.008  10.1128/mSphere.00046-17 |
| Crohn’s disease or ulcerative colitis | GF 129S6/SvEv background wildtype and *Il-10*-deficient mice | Donor: 5 adult patients with active Crohn’s disease (4 donors) or ulcerative colitis (1 donor). Methodology: oral gavage. | The host inflammation promotes aggressive resident bacteria, which further drives a feed-forward process of dysbiosis exacerbated by gut inflammation. | (Gray et al., 2024) | 10.1186/s40168-024-01857-2 |
| Crohn’s disease | ATG16L1 T300A knock-in mice（C57BL/6J） | Donor: Crohn’s disease patients.  Methodology: 25 mg (100 μl) was orally instilled, followed by 12.5 mg (50 μl) placed on the anus and 12.5 mg (50 μl) placed on the back fur of each mouse. | ATG16L1 T300A mice exhibited elevated *Bacteroides ovatus* and increased Th1/Th17 cells before disease onset, indicating that the variant drives dysbiosis and immune infiltration before symptom manifestation. | (Lavoie et al., 2019) | 10.7554/eLife.39982 |
| Colorectal cancer | GF male C57BL/6 mice | Donor: 3 healthy individuals and 3 patients found to harbor carcinomas.  Methodology: oral gavage. Three weeks after inoculation, mice received a single intraperitoneal injection of azoxymethane. Five days later, mice were subjected to the first of three five-day rounds of 2% dextran sulfate sodium administered *ad libitum* in the drinking water. | Chemically induced Colorectal cancer in colonized mice yielded a variable tumor burden that correlated with the mice’s baseline microbiome composition, not with the donors’ cancer status. | (Baxter et al., 2014) | 10.1186/2049-2618-2-20 |
| Colorectal cancer | GF *Apc^Min/+^* mice(C57BL/6J) | Donor: colorectal cancer patients who ate  Yi-Yi-Fu-Zi-Bai-Jiang-San (YYFZBJS). Methodology: oral gavage. | The stool from YYFZBJS volunteers transplantation reduced intestinal tumor burden, modulated inflammatory gene expression, and lowered Tregs in intestinal lymphatics and mesenteric lymph nodes to inhibit colorectal cancer cell proliferation. | (Sui et al., 2020) | 10.1186/s12964-020-00596-9 |
| Melanoma | GF female/male C57BL/6 mice | Donor: melanoma patients who responded (R) or did not respond (NR) to anti‑PD‑1 therapy. Methodology: oral gavage into mice for 3 doses over a week. Followed by a break of a week to allow microbiome establishment. Mice were then injected with the BRAFV600E/PTEN-/- (BP) syngeneic tumor cell line (Day 14), and animals were treated with anti-PD-L1 monoclonal antibody. | Mice receiving R‑derived microbiota showed significantly slower tumor growth, higher survival, and increased intratumoral CD8⁺ T‑cell infiltration after anti‑PD‑1 treatment, whereas NR‑colonized mice resembled control (no‑response) mice. The phenotype was transferable and correlated with microbial diversity. | (Gopalakrishnan et al., 2018; Matson et al., 2018) | 10.1126/science.aan4236  10.1126/science.aao3290 |
| Pancreatic Cancer | GF male C57BL/6J mice | Donor: 5 pancreatic cancer patients and healthy volunteers.  Methodology: oral gavage on days 0, 2 and 6. | Mice receiving fecal material from pancreatic‑cancer patients and healthy donors showed comparable body‑weight gain and food intake, but those receiving the pancreatic‑cancer microbiota exhibited reduced visceral‑fat accumulation. | (Genton et al., 2021) | 10.3389/fcimb.2021.752889 |
| Pancreatic‑ductal adenocarcinoma | GF male C57BL/6 mice | Donor: pancreatic‑ductal adenocarcinoma patients who responded (R) or did not respond (NR) to standard chemotherapy (gemcitabine‑based regimens). Methodology: single oral gavage. | Mice colonized with R-derived microbiota displayed markedly enhanced chemotherapy efficacy: smaller pancreatic tumor volumes, reduced tumor‑growth rate, and prolonged survival compared with NR‑colonized or GF controls. | (Tintelnot et al., 2023) | 10.1038/s41586-023-05728-y |
| Preterm infant | Pregnant GF C57BL/6 J mice | Donor: an infant with low or high growth.  Methodology: oral gavage. | Mice receiving the low‑growth microbiota displayed reduced neuronal markers (NeuN, neurofilament‑L) and myelination marker (MBP), increased neuroinflammation (Nos1), and altered IGF‑1 signaling. These changes mirror the impaired neurodevelopment observed in preterm infants with poor growth. | (Lu et al., 2018) | 10.1038/s41598-018-23692-w |
| Preterm infant and Necrotizing enterocolitis | Pregnant GF C57BL/6 J mice | Donor: 2 preterm infants who later developed necrotizing enterocolitis and 2 healthy term infants.  Methodology: oral gavage. | Mice showed a leaky gut with reduced occludin/ZO‑1 and increased NF‑κB signaling, exhibited anxiety‑like behavior and reduced locomotion, had impaired contextual memory, displayed delayed myelination and lower fractional anisotropy in key brain regions, and presented altered brain metabolites, recapitulating the neurodevelopmental deficits seen in preterm infants with necrotizing enterocolitis. | (Lu et al., 2023) | 10.3390/microorganisms11051131 |
| Parkinson's Disease | GF C57BL/6 J male mice that carry the Thy1-αSyn transgene | Donor: parkinson’s disease patients and matched healthy controls. Methodology: oral gavage. | Mice receiving fecal material from parkinson’s disease patients displayed pronounced motor impairments (beam traversal, pole descent, hind‑limb clasping) and exacerbated gastrointestinal deficits. | (Sampson et al., 2016) | 10.1016/j.cell.2016.11.018 |

DOI, digital object identifier. GF, germ‑free. OGTT: oral glucose tolerance test. AUC: area under the curve.
